# Elevations in growth hormone and glucagon-like peptide-2 levels on admission are associated with increased mortality in trauma patients

**DOI:** 10.1186/s13049-016-0310-8

**Published:** 2016-10-04

**Authors:** Matthew P. Rowan, Darrick J. Beckman, Julie A. Rizzo, Claire L. Isbell, Christopher E. White, Stephen M. Cohn, Kevin K. Chung

**Affiliations:** 1 United States Army Institute of Surgical Research, 3698 Chambers Pass, JBSA, Fort Sam Houston, San Antonio, TX 78234 USA; 2 Brooke Army Medical Center, 3855 Roger Brooke Drive, JBSA, Fort Sam Houston, San Antonio, TX 78234 USA; 3 Uniformed Services University of the Health Sciences, 4301 Jones Bridge Rd # A3007, Bethesda, MD 20814 USA; 4 Baylor Scott and White Memorial Hospital, 2401 S. 31st St, Temple, TX 76502 USA; 5 Staten Island University Hospital, 475 Seaview Ave, Staten Island, NY 10305 USA

**Keywords:** Biomarker, Burn, Hormone, Hypermetabolism, Trauma

## Abstract

**Background:**

Burn and trauma patients present a clinical challenge due to metabolic derangements and hypermetabolism that result in a prolonged catabolic state with impaired healing and secondary complications, including ventilator dependence. Previous work has shown that circulating levels of growth hormone (GH) are predictive of mortality in critically ill adults, but few studies have examined the prognostic potential of GH levels in adult trauma patients.

**Methods:**

To investigate the utility of GH and other endocrine responses in the prediction of outcomes, we conducted a prospective, observational study of adult burn and trauma patients. We evaluated the serum concentration of GH, insulin-like growth factor 1 (IGF-1), IGF binding protein 3 (IGFBP-3), and glucagon-like peptide-2 (GLP-2) weekly for up to 6 weeks in 36 adult burn and trauma patients admitted between 2010 and 2013.

**Results:**

Non-survivors had significantly higher levels of GH and GLP-2 on admission than survivors.

**Discussion:**

This study demonstrates that GH has potential as a predictor of mortality in critically ill trauma and burn patients. Future studies will focus on not only the role of GH, but also GLP-2, which was shown to correlate with mortality in this study with a goal of offering early, targeted therapeutic interventions aimed at decreasing mortality in the critically injured.

**Conclusions:**

GH and GLP-2 may have clinical utility for outcome prediction in adult trauma patients.

**Electronic supplementary material:**

The online version of this article (doi:10.1186/s13049-016-0310-8) contains supplementary material, which is available to authorized users.

## Background

Severe trauma, burns, and critical illness affect millions of people every year and present a significant challenge to clinicians due to prolonged stays and metabolic derangements. Burns and other major traumatic injuries lead to a severe hypermetabolic response with elevated resting energy expenditure, insulin resistance, altered substrate use, elevations in protein synthesis and breakdown, and a negative nitrogen balance despite adequate nutrition [1]. The resulting catabolic state is characterized by impaired wound healing, muscle weakness, immobility, and prolonged ventilator dependence [2–5]. Current treatment approaches focus on managing the consequences of hypermetabolism through supportive therapy, but new approaches and advancements are needed to treat or attenuate the post-trauma hypermetabolic response.

The growth hormone (GH) axis is a key metabolic regulator that holds several potential targets for therapeutic intervention [5, 6]. Activation of the hypothalamus causes the release of growth hormone releasing hormone, which then activates the pituitary to release GH that acts on a number of targets, such as fat and liver, to increase the concentration of glucose and free fatty acids and the production of additional hormones, including insulin-like growth factor 1 (IGF-1) [7], which is highly protein bound, most commonly to IGF binding protein 3 (IGFBP-3), but when free activates the Akt signaling pathway to induce cell proliferation and inhibit apoptosis. The GH axis response to critical illness is biphasic, with acute and chronic phases [8, 9]. The acute phase (5–10 days) is marked by an actively secreting anterior pituitary with an increase in GH levels and paradoxically decreased IGF-1 and IGFBP-3, secondary to GH resistance [9, 10]. Transition to the chronic phase is marked by a decrease in GH secretion, persistently low IGF-1 and IGFBP-3, and continued protein catabolism [9, 11–13].

Therapeutic interest has focused on targeting the chronic phase of the post-injury endocrine response, as ongoing catabolism is associated with increased morbidity and mortality [6, 14]. IGF-1 administration has been shown to improve patient outcomes, such as wound healing, muscle protein synthesis, and immune function [15–18]. Recombinant human growth hormone (rhGH) supplementation results in increased IGF-1 and positive nitrogen balance in patients with burns [19, 20], post-operative patients [21, 22], and the critically ill [23]. Additional studies with rhGH have shown improved morbidity and mortality in burn patients, including adolescents [24–30], and a recent Cochrane review of available randomized, controlled clinical trials of GH in patients with large burns concluded that GH treatment results in accelerated healing in both burn wounds and donor sites [31]. However, a large, multi-center, double-blind, placebo-controlled study showed that rhGH administration is associated with an increased risk of mortality in critically ill, non-trauma patients [32], suggesting that the target patient population may be an important caveat for rhGH intervention.

A clearer understanding of the timing and magnitude of the endocrine response to severe trauma would increase the likelihood of identifying targets for therapeutic intervention in order to minimize the impact of the hypermetabolic response. Few studies have examined the prognostic value of the GH axis in adult critically ill patients. Recent work showed no significant differences in GH levels among critically ill patients [33] whereas others have demonstrated that GH levels on admission were higher in non-survivors and were directly correlated with severity of sepsis and Acute Physiology and Chronic Health Evaluation II (APACHE II) score. Low IGF-1 levels were also associated with higher mortality [34], however IGFBP-3 was not correlated with APACHE II scores or mortality [35]. Furthermore, GH levels remained elevated after 24 h and at discharge or death in non-survivors and, along with IL-6 levels and APACHE II score, was an independent predictor of mortality [35]. To evaluate the prognostic value of other endocrine hormones, this study examined changes in the concentrations of GH, IGF-1, IGFBP-3, and glucagon-like peptide-2 (GLP-2) in adult burn and trauma patients with severe injury.

## Methods

### Subjects

This prospective, observational study was conducted under a protocol reviewed and approved by the Brooke Army Medical Center Institutional Review Board and in accordance with the approved protocol. Adults (≥18 years old) were eligible for the study if they were admitted to the Intensive Care Unit (ICU) at San Antonio Military Medical Center (trauma) or United States Army Institute of Surgical Research (burn) with a severe injury, defined as trauma with injury severity score (ISS) >15 or burn covering ≥20 % of the total body surface area (TBSA). Patients with known endocrine disorders other than diabetes mellitus were excluded. Delayed consent was used for subject enrollment in accordance with the approved study procedures. Additionally, five healthy subjects were selected as controls to establish uninjured values. Subject demographics, injury information, and clinical outcomes data were collected.

### Sample collection

Blood samples were collected from existing central venous catheters, arterial lines, or intravenous lines for analysis of GH, IGF-1, IGFBP-3, and GLP-2 levels. A total of 4 mL of blood was collected twice (morning and evening) on admission (day 1) and on days 7, 14, 21, 28, 35, and 42, when possible. Samples were collected within 24 h of admission on all patients and for patients who proceeded to the operating room on the day of admission, samples were collected prior to the operation. Blood samples were centrifuged (1000 *g*, 15 min, room temperature) and the serum was separated within 90 min of collection and aliquoted for storage at −80 °C until analysis.

### Hormone analysis

Serum samples were analyzed with commercially available ELISA kits for GH (DGH00, R&D Systems, Minneapolis, MN), IGF-1 (DG100, R&D Systems, Minneapolis, MN), IGFBP-3 (DBG300, R&D Systems, Minneapolis, MN), and GLP-2 (YK141, Yanaihara Institute, Shizuoka, Japan) according to manufacturer instructions.

### Statistical analysis

Continuous variables were analyzed using either the Student’s *t*-test or Wilcoxon signed-rank test, based on the results of the Shapiro-Wilks test of normality. Log-transformations were considered for right-skewed data, and log-normally distributed data is presented as geometric mean with 95 % confidence interval. Variables that passed or failed the test for normality are presented as mean (± standard deviation) or median (interquartile range), respectively. Categorical variables were compared using Chi-squared, Mann Whitney (when sample sizes were unequal), or Fisher’s exact tests, as appropriate. Linear regression was performed to determine an association between hormone levels over time and other factors. P-values and overall Pearson’s correlation coefficient were reported for all regression models. Statistical significance was accepted at *p* < 0.05. All data were analyzed with SAS 9.1 (SAS, Cary, NC).

## Results

A total of 36 subjects were enrolled in this study. A summary of the different injuries are shown in Table 1; more detailed information on the subject demographics, detailed injury information and clinical outcomes are available (Additional file 1: Table S1). Subjects ranged from 18 to 74 years old (median 31.5) and were predominantly male (81 %) civilians (81 %). Body mass index values ranged from 19.4 to 42.2 (median 28.6). Subjects were most commonly injured in automotive crashes (14 motor vehicle and 3 motorcycle), assaults with or without gunshot wounds (5), explosions/blasts with or without burns (5), and burns (5). Average ISS was 29 ± 10.2 (range 9–57, median 27) and average APACHE II score was 22.7 ± 9.8 (range 6–44, median 25). Subjects averaged 26.1 days in the hospital (range 4–124, median 17), 14.9 days in the ICU (range 1–62, median 11), 9.3 days on a ventilator (range 0–43, median 6), and had an overall in-hospital mortality of 19 %.

**Table 1:** Injury information. Polytrauma patients experienced more than one type of injury, therefore the total number of injuries is greater than the number of patients

Type of injury	Incidence (#)
Traumatic brain injury	16
Solid organ injury	11
Long bone fracture	8
Spinal cord injury/spinal fracture	10
Pelvic fracture	3
Rib fracture(s)	5
Burn	6
Other^a^	13

^a^includes fractures other than long bones, pelvis or rib, pneumothorax, hollow viscous injuries and diaphragm injuries

**Fig. 1:**
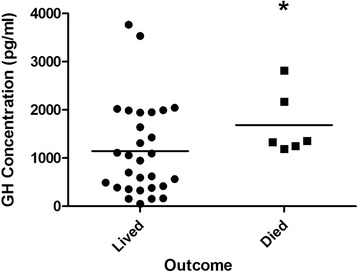
Growth hormone (GH) concentration on admission (day 1) in survivors and non-survivors of trauma. *, *p* < 0.05 by Mann-Whitney

**Fig. 2:**
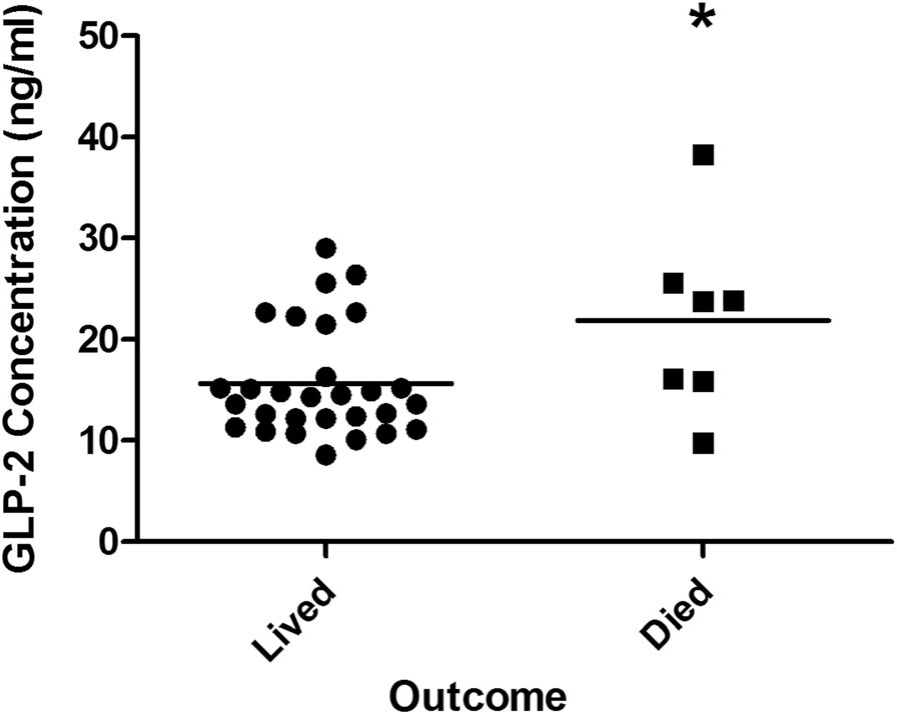
Glucagon-like peptide-2 (GLP-2) concentration on admission (day 1) in survivors and non-survivors of trauma. *, *p* < 0.05 by Mann-Whitney

**Fig. 3:**
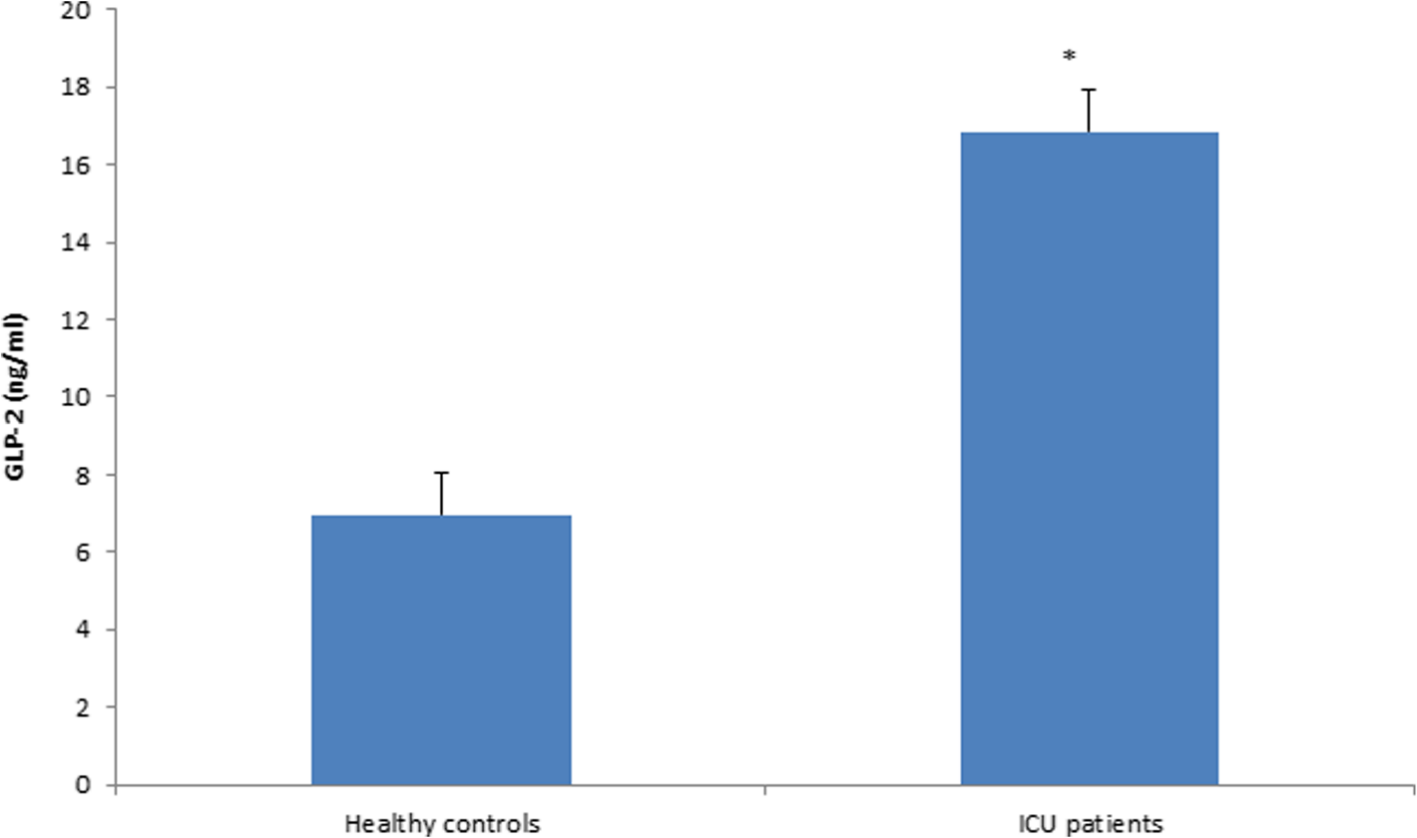
GLP-2 concentration on admission (day 1) in patients compared to healthy controls. *, *p* < 0.05 by Mann-Whitney

**Fig. 4:**
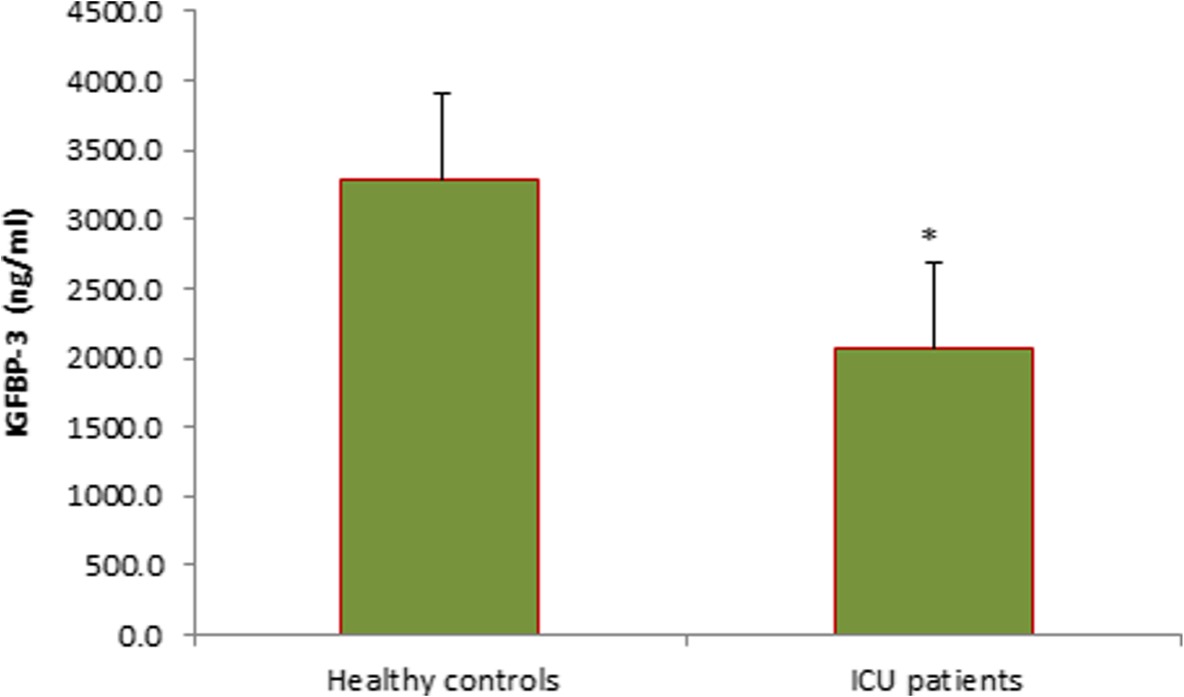
IGFBP-3 concentration on admission (day 1) in patients compared to healthy controls. *, *p* < 0.05 by Mann-Whitney

Samples were collected from all 36 subjects upon admission (day 1) but due to factors such as length of stay, study withdrawal, hospital discharge and mortality, weekly samples could not be collected for all subjects. Data was available from 21, 12, 9, 6, 4, and 2 subjects on days 7, 14, 21, 28, 35, and 42, respectively. Due to hormone variability and reduced power over time, no temporal trends were detected in any of the hormones evaluated. Likewise, subgroup analysis comparing burn patients with patients positive for traumatic brain injury (TBI) and non-TBI trauma patients revealed no significant correlations in hormone levels (Table 2). Age was inversely correlated with average levels of GH (*p* = 0.016, *R* ^2^ = 16 %), IGF-1 (*p* = 0.0001, *R* ^2^ = 35 %), and IGFBP-3 (*p* = 0.003, *R* ^2^ = 23 %), and males had significantly higher levels of GLP-2 (*p* = 0.049) and IGF-1 (*p* = 0.01). Consistent with previous reports [35], non-survivors had significantly higher GH levels on admission than survivors (Fig. 1, *p* = 0.038). Furthermore, higher GLP-2 levels on admission were associated with higher mortality (Fig. 2, *p* = 0.016). As expected, ISS and APACHE II scores were significantly associated with increased mortality (*p* = 0.035 and *p* = 0.004, respectively). Comparison of burn/trauma ICU patients to healthy controls showed that, on average, ICU patients had higher levels of GLP-2 (Fig. 3, *p* < 0.0001) but lower levels of IGFBP-3 (Fig. 4, *p* = 0.011).

**Table 2:** Mean hormone levels comparing burn patients to non-burned trauma patients positive for TBI with non-TBI trauma patients

Hormone	Burn	TBI	Non-TBI trauma
GH^a^	3.22	2.76	2.84
GLP-2^b^	1.16	1.23	1.15
IGF-1^c^	2.06	1.77	1.87
IGFBP-3^d^	3.41	3.24	3.27

^a^ *p* = 0.0625, *R* ^2^ = 10.9 %

^b^ *p* = 0.293, *R* ^2^ = 21.6 %

^c^ *p* = 0.052, *R* ^2^ = 10.9 %

^d^ *p* = 0.252, *R* ^2^ = 8.85 %

## Discussion

Severe traumatic injuries, including burns, are a unique, complex challenge to clinicians. Patients often demonstrate major alterations in metabolic and endocrine responses that induce a host of physiological complications that often require intensive care and multiple organ support. In addition to established predictors of patient outcome, such as ISS and APACHE II scores, prognostic indicators of survival or disease progression could allow for earlier targeted intervention and improved patient outcomes. Furthermore, the ability to triage patients and focus care, when resources are limited, based on a combination of injury severity and outcome predictors could improve survival and functional recovery. In this study, the levels of GH, IGF-1, IGFBP-3, and GLP-2 were tracked in 36 burn and trauma patients over a 6 week period and compared with outcomes data (hospital days, ICU days, ventilator days, and survival). Non-survivors had significantly higher GH and GLP-2 levels, ISS and APACHE II scores on admission, and average GLP-2 levels than survivors. When compared with healthy controls, ICU patients had higher levels of GLP-2 and IGFBP-3.

Few studies have evaluated the prognostic potential of GH, IGF-1, IGFBP-3, or GLP-2 in trauma patients. A recent study of 103 critically ill patients found that survivors had significantly lower GH levels on admission than non-survivors, with or without sepsis, and that GH levels remain elevated in non-survivors after 24 h and at discharge or death [35]. Similarly, dichotomizing patients into low (<25) and high (≥25) risk based on APACHE II score revealed elevated GH levels in high risk patients. In fact, elevated GH at admission was identified as an independent predictor of mortality, and improved the prognostic accuracy of the APACHE II score when used in combination. These data are in agreement with the elevated GH levels seen in the 36 burn and trauma patients in the present study, and increase the potential of GH as an indicator of mortality in critically ill patients.

Therapeutic interest in attenuating the hypermetabolic response after injury has led to evaluating the effect of different therapies, such as insulin [9, 36], propranolol [37], and IGF-1 [15–18], which has been shown to improve patient outcomes such as wound healing, muscle protein synthesis, and immune function. Significant differences in IGF-1 levels between survivors and non-survivors were not detected in this study, but this is likely because of several limitations, including reduced power from small sample size and patient attrition throughout the 6 week study. The high patient attrition rate also impairs the ability to reliably correlate the measured values with parameters such as length of stay, ICU days, ventilator days and perhaps even survival. Studies have also explored supplementation with rhGH which has been shown to increase IGF-1 levels, accelerate healing, and improve morbidity and mortality in burn patients in several studies [31].

The present study is in agreement with previous work [35] that shows higher GH levels in critically ill trauma patients than uninjured controls. Furthermore, GH levels on admission are correlated with outcome (Fig. 1) and serve as an independent predictor for mortality [35], which suggests that GH supplementation could be unnecessary or potentially dangerous in some critically ill patients. This could at least partly explain the results of a large multi-center trial that showed an increased risk of mortality with rhGH administration in non-trauma patients [32]. Additional side effects of GH supplementation have been noted to include hyperglycemia [31], sodium retention leading to an increase in extracellular water [35], hypercalcemia and hypercalciuria [38, 39]. More work is needed to identify the target patient population for rhGH administration in order to maximize any potential benefit and minimize potential complications of GH therapy in critically ill patients.

## Conclusion

The treatment of severe trauma, burns, and critical illness remains a challenge to clinicians, but the ability to predict patient outcomes has impacted patient care to allow for early intervention and improved outcomes. New markers are needed to allow for more accurate, earlier predictions of patient outcome, and to improve the delivery of precision medicine. To our knowledge, this is the first study to identify GLP-2 as a novel prognostic indicator in trauma patients. Additional work is needed in larger patient populations across multiple centers to increase the impact of GLP-2, GH, and other clinically relevant hormones as potential indicators of clinical outcome.

## Additional file

Additional file 1: Table S1. Patient demographics, injury information, and clinical outcomes. APACHE II = Acute Physiology and Chronic Health Evaluation II, Civ = civilian, GSW = gunshot wound, IED = improvised explosive device, ISS = Injury Severity Score, MCC = motorcycle crash, Mil = military, MVC = motor vehicle collision, N = no, TBI = traumatic brain injury, TBSA = total body surface area, Y = yes. (DOCX 23 kb)

## Abbreviations

APACHE II: Acute Physiology and Chronic Health Evaluation II
GH: Growth hormone
GLP-2: Glucagon-like peptide 2
ICU: Intensive care unit
IGF-1: Insulin-like growth factor 1
IGFBP-3: Insulin-like growth factor binding protein 3
IL: Interleukin
ISS: Injury severity score
rHGH: Recombinant human growth hormone
TBI: Traumatic brain injury
TBSA: Total body surface area
USAMRMC: United States Army Medical Research and Materiel Command
