# Supplementary material for: Elevations in growth hormone and glucagon-like peptide-2 levels on admission are associated with increased mortality in trauma patients
Source: Scand J Trauma Resusc Emerg Med. 2016 Oct 4;24:119. doi: 10.1186/s13049-016-0310-8 (PMC5050752; doi:10.1186/s13049-016-0310-8)
Supplement: Additional file 1: Table S1. — Patient demographics, injury information, and clinical outcomes. APACHE II = Acute Physiology and Chronic Health Evaluation II, Civ = civilian, GSW = gunshot wound, IED = improvised explosive device, ISS = Injury Severity Score, MCC = motorcycle crash, Mil = military, MVC = motor vehicle collision, N = no, TBI = traumatic brain injury, TBSA = total body surface area, Y = yes. (DOCX 23 kb) [file 13049_2016_310_MOESM1_ESM.docx]

| # | Injury Information | Burn  (Y/N) | TBI  (Y/N) | How Injury Received | Age | Sex | Civ/Mil | ISS | APACHE II | Died (Y/N) | ICU Days | Hospital days | Ventilator days |
| --- | --- | --- | --- | --- | --- | --- | --- | --- | --- | --- | --- | --- | --- |
| 1 | C1 fracture, liver laceration, coronary artery dissection | N | N | MVC | 56 | F | Civ | 35 | 8 | N | 13 | 23 | 0 |
| 2 | Splenic/hepatic/renal lacerations, humerus fracture | N | N | MVC | 29 | F | Civ | 33 | 35 | N | 6 | 14 | 2 |
| 3 | Subdural hemorrhage, skull fracture | N | Y | MVC | 58 | M | Civ | 29 | 9 | N | 4 | 4 | 0 |
| 4 | Skull fracture, epidural hematoma | N | Y | MVC | 24 | M | Civ | 35 | 21 | N | 11 | 14 | 7 |
| 5 | Epidural hematoma, rib fractures, hip fracture | N | Y | Trauma | 50 | M | Civ | 34 | 13 | N | 4 | 12 | 0 |
| 6 | Liver laceration, hemothorax | N | N | GSW | 29 | M | Civ | 20 | 6 | N | 2 | 5 | 0 |
| 7 | Spinal cord injury | N | N | MVC | 24 | M | Civ | 18 | 18 | N | 43 | 43 | 43 |
| 8 | 30% TBSA burn | Y | N | Burn | 54 | M | Civ | 16 | 16 | N | 9 | 36 | 2 |
| 9 | Splenic laceration, spinal fractures | N | N | MVC | 29 | M | Civ | 34 | 28 | N | 3 | 9 | 2 |
| 10 | Femur fracture, pelvic fracture, bladder rupture | N | N | MCC | 21 | M | Mil | 22 | 8 | N | 6 | 22 | 1 |
| 11 | Rib fractures, hand fractures | N | N | MVC | 42 | M | Civ | 13 | 12 | N | 4 | 8 | 0 |
| 12 | Subdural hematoma, facial fractures, pneumothorax | N | Y | Blast | 49 | M | Civ | 34 | 29 | N | 9 | 14 | 3 |
| 13 | Pelvic fracture, spinal fracture, sternal fracture | N | N | MVC | 24 | F | Civ | 27 | 6 | N | 1 | 7 | 0 |
| 14 | Spinal fracture, rib fractures, hemothorax | N | N | Assault | 33 | M | Civ | 29 | 17 | N | 16 | 21 | 6 |
| 15 | Rib fractures, spinal fractures, long bone fractures | N | N | MVC | 31 | M | Civ | 22 | 20 | N | 7 | 26 | 3 |
| 16 | 56% TBSA burn, inhalation injury | Y | N | Burn | 37 | M | Mil | 41 | 25 | Y | 41 | 41 | 41 |
| 17 | Epidural hematoma, skull fracture, pneumothorax | N | Y | MVC | 44 | F | Mil | 25 | 19 | N | 27 | 27 | 8 |
| 18 | Hemopneumothorax | N | N | GSW | 39 | M | Civ | 27 | 15 | N | 4 | 8 | 1 |
| 19 | Subdural hematoma, facial fractures | N | N | MVC | 21 | M | Civ | 19 | 8 | N | 7 | 11 | 4 |
| 20 | Splenic/liver lacerations, femur fractures, intraparenchymal hemorrhage | N | Y | MVC | 27 | M | Civ | 36 | 39 | Y | 11 | 11 | 11 |
| 21 | Dismounted IED, 29% TBSA burn, splenic laceration, facial fractures | Y | N | Blast | 32 | M | Mil | 57 | 26 | N | 49 | 92 | 22 |
| 22 | Mounted IED, rib fractures, spinal fractures, long bone fractures, closed head injury | N | Y | Blast | 24 | M | Mil | 29 | 23 | N | 9 | 26 | 6 |
| 23 | Subdural/epidural hematomas, facial fractures | N | Y | GSW | 18 | M | Civ | 25 | 30 | N | 10 | 15 | 9 |
| 24 | Subdural/subarachnoid hemorrhage, skull fracture | N | Y | GSW | 34 | M | Civ | 26 | 32 | Y | 12 | 12 | 12 |
| 25 | Intraparenchymal hemorrhage, skull/facial fractures | N | Y | MVC | 24 | M | Civ | 34 | 28 | N | 20 | 40 | 17 |
| 26 | 26% TBSA burn | Y | N | Burn | 18 | M | Civ | 9 | 24 | N | 12 | 21 | 0 |
| 27 | Spinal fracture, pelvic fracture, rib fractures, pneumothoraces | N | N | MCC | 61 | M | Civ | 34 | 35 | Y | 29 | 44 | 22 |
| 28 | Dismounted IED, traumatic leg amputation, soft tissue defects | N | N | Blast | 25 | M | Mil | 21 | 25 | N | 8 | 39 | 4 |
| 29 | Dismounted IED, intraparenchymal hemorrhage, colon laceration, traumatic leg amputation | N | Y | Blast | 23 | M | Mil | 34 | 25 | Y | 13 | 13 | 7 |
| 30 | Subarachnoid hemorrhage | N | Y | Trauma | 62 | F | Civ | 16 | 28 | Y | 13 | 13 | 13 |
| 31 | Spinal fractures, colon/splenic lacerations, arm fractures, subarachnoid hemorrhage | N | Y | MCC | 21 | M | Civ | 41 | 44 | N | 22 | 124 | 11 |
| 32 | Subdural hematoma, spinal fracture | N | Y | Trauma | 74 | F | Civ | 25 | 30 | N | 11 | 18 | 8 |
| 33 | Liver laceration, subdural/intraparenchymal hemorrhage, diaphragm rupture | N | Y | MVC | 31 | M | Civ | 48 | 34 | Y | 12 | 12 | 12 |
| 34 | 40% TBSA burn | Y | N | Burn | 59 | M | Civ | 25 | 28 | N | 21 | 32 | 13 |
| 35 | Subdural hematoma | N | Y | Trauma | 70 | F | Civ | 9 | 26 | N | 7 | 16 | 5 |
| 36 | 31% TBSA burn | Y | N | Burn | 57 | F | Civ | 25 | 26 | N | 62 | 66 | 41 |

Supplemental Table 1. Patient demographics, injury information, and clinical outcomes. APACHE II = Acute Physiology and Chronic Health Evaluation II, Civ = civilian, GSW = gunshot wound, IED = improvised explosive device, ISS = Injury Severity Score, MCC = motorcycle crash, Mil = military, MVC = motor vehicle collision, N = no, TBI = traumatic brain injury, TBSA = total body surface area, Y = yes.
